# Tracking emotions in the brain – Revisiting the Empathic Accuracy Task

**DOI:** 10.1016/j.neuroimage.2018.05.080

**Published:** 2018-09

**Authors:** Nuria K. Mackes, Dennis Golm, Owen G. O'Daly, Sagari Sarkar, Edmund J.S. Sonuga-Barke, Graeme Fairchild, Mitul A. Mehta

**Affiliations:** a Department of Child and Adolescent Psychiatry, Institute of Psychiatry, Psychology and Neuroscience, King's College London, De Crespigny Park, SE5 8AF, London, UK; b Department of Neuroimaging, Institute of Psychiatry, Psychology and Neuroscience, King's College London, De Crespigny Park, SE5 8AF, London, UK; c Department of Psychology, University of Bath, 10 West, BA2 7AY, Bath, UK

**Keywords:** Empathy, Emotion, Social cognition, fMRI, Ecological validity

## Abstract

Many empathy tasks lack ecological validity due to their use of simplistic stimuli and static analytical approaches. Empathic accuracy tasks overcome these limitations by using autobiographical emotional video clips. Usually, a single measure of empathic accuracy is computed by correlating the participants' continuous ratings of the narrator's emotional state with the narrator's own ratings.

In this study, we validated a modified empathic accuracy task. A valence-independent rating of the narrator's emotional intensity was added to provide comparability between videos portraying different primary emotions and to explore changes in neural activity related to variations in emotional intensity over time. We also added a new neutral control condition to investigate general emotional processing. In the scanner, 34 healthy participants watched 6 video clips of people talking about an autobiographical event (2 sad, 2 happy and 2 neutral clips) while continuously rating the narrator's emotional intensity.

Fluctuation in perceived emotional intensity correlated with activity in brain regions previously implicated in cognitive empathy (bilateral superior temporal sulcus, temporoparietal junction, and temporal pole) and affective empathy (right anterior insula and inferior frontal gyrus). When emotional video clips were compared to neutral video clips, we observed higher activity in similar brain regions. Empathic accuracy, on the other hand, was only positively related to activation in regions that have been implicated in cognitive empathy.

Our modified empathic accuracy task provides a new method for studying the underlying components and dynamic processes involved in empathy. While the task elicited both cognitive and affective empathy, successful tracking of others' emotions relied predominantly on the cognitive components of empathy. The fMRI data analysis techniques developed here may prove valuable in characterising the neural basis of empathic difficulties observed across a range of psychiatric conditions.

## Highlights

• Activity in affective and cognitive empathy related regions during emotional videos.
• Activity in similar regions related to changes in perceived emotional intensity.
• Only regions implicated in cognitive empathy were associated with empathic accuracy.
• No difference between video clips that did and did not elicit affect sharing.
• Empathic accuracy related to self-rated perspective-taking but not empathic concern.

## Introduction

Empathy, has been defined as “an emotional response [… which] is similar to one's perception […] and understanding […] of the stimulus emotion, with recognition that the source of the emotion is not one's own.” (Cuff et al., 2016, page 150). Empathy is crucial for successful social interaction as it allows the individual to predict others' actions, emotions and intentions (Bernhardt and Singer, 2012). Deficits in empathic processing have been reported in psychiatric disorders such as autism spectrum disorder (ASD), schizophrenia, borderline personality disorder and bipolar disorder (Gonzalez-Liencres et al., 2013). Identifying the neural substrates of empathy in healthy populations is important for understanding conditions that are characterised by empathic difficulties. In neuroscience, the concept of empathy is considered to include separate affective (sharing others' emotion) and cognitive (understanding others' emotion) components (for example, Tousignant et al., 2017; Lockwood, 2016). Previous research has identified distinct clusters of brain regions involved in affective empathy: medial/anterior cingulate cortex (MCC, ACC), anterior insula (AI) (Fan et al., 2011; Lamm et al., 2011), and supplementary motor area (SMA) (Lamm et al., 2011). Within the broader domain of social cognition, cognitive empathy overlaps with the affective component of Theory of Mind (ToM) or mentalising, namely the capacity to infer other people's thoughts, emotions and intentions without necessarily sharing them (Wellman et al., 2001). A recent meta-analysis of 144 fMRI studies using ToM tasks (Molenberghs et al., 2016) identified the medial prefrontal cortex (mPFC), medial orbitofrontal cortex (mOFC), ACC, precuneus, temporal pole (TP), posterior superior temporal gyrus (pSTS) and temporoparietal junction (TPJ) and inferior frontal gyrus (IFG) as key regions for mentalising.

However, prior research on the neural mechanisms of empathy has often lacked ecological validity. Studies have often used simplistic stimuli that differ greatly from the complex cues that individuals have to process in real-life situations (Zaki and Ochsner, 2009, 2012). Moreover, most studies focus on empathy for pain, while only a few studies have evaluated other emotions (e.g. disgust, happiness, sadness; Fan et al., 2011; Walter, 2011). In addition, empathy has mostly been operationalised as a static trait (Cuff et al., 2016). However, in the real world empathy fluctuates dynamically (Ashar et al., 2017). These fluctuations can happen spontaneously because of changes in internal state or in response to shifts in external circumstances, such the emotional intensity and expressivity of others.

In the current study, we addressed these limitations of previous research by modifying an existing paradigm, the Empathic Accuracy Task (EAT; Zaki et al., 2009), that incorporates more naturalistic stimuli and reflects the dynamic nature of empathy. Participants (perceivers) watch video clips in which another person (target) describes an emotional autobiographical event. Perceivers continuously rate the target's emotion while watching the clips (via button pressing). The EAT measures how *accurately* the perceiver infers changes in the target's emotional states by correlating the perceiver's ratings with the target's ratings of their own emotions (see Zaki et al., 2009 for a detailed description). Zaki et al. (2009) found that empathic accuracy was associated with higher activation in both affective (i.e. inferior parietal lobule (IPL)) and cognitive (i.e. mPFC) empathy networks. In a recent study of adolescents, empathic accuracy related positively to activation in cognitive empathy or mentalising regions (mPFC, TPJ, STS) and negatively to activation in regions implicated in affective empathy (IPL, ACC, AI; Kral et al., 2017).

In the current study, new video clips were created and the EAT was modified in the following important ways: First, video clips depicted discrete primary emotions (happy, sad, angry, frightened) and participants rated changes in the targets' emotional intensity (instead of valence) to ensure comparability across different emotions and higher construct validity. Second, we introduced well-matched neutral video clips that acted as a control condition. In this condition, targets described their bedroom. This control condition allowed us to examine the neural correlates of emotion processing irrespective of empathic accuracy. Third, as empathy is a dynamic process, perceivers need to be able to continuously identify changes in the intensity of the target's emotional state. We therefore utilised an analysis approach that tracked changes in the target's emotional intensity throughout each video clip, in addition to deriving a single index of empathic accuracy (averaged across the clip). Fourth, we included ratings from participants regarding how they felt after watching each video to gain a better understanding of how the neural correlates of EA are influenced by cognitive and affective empathy. Finally, to validate the EAT, we related task performance to self-reported trait empathy and IQ as well as acquiring a normative data set with participants who completed the EAT outside of the scanner.

The aim of this study was to validate a modified version of the Empathic Accuracy Task, using a staged analysis approach which replicates analyses presented previously in the literature, but which also included additional comparisons. First, we contrasted the blood-oxygen-level dependent (BOLD) responses to emotional and neutral clips to explore correlates of complex and multi-sensory emotional processing during extended clips rather than single emotional images. Second, we validated our emotional intensity rating scale by analysing the neural correlates of intra-individual variations in empathic accuracy. Third, we explored neural correlations with variations in perceived emotional intensity over time, thus capitalising on the availability of continuous ratings throughout each video clip.

Given the results of prior neuroimaging studies of empathy and mentalising, we had the following hypotheses:

(1) At the group level, increased BOLD responses would be observed in brain regions previously linked to empathy and mentalising when participants watched targets describe emotional versus neutral events.
(2) There would be positive correlations between intra-individual variations in empathic accuracy and BOLD responses in these regions.
(3) We predicted positive correlations between fluctuations in perceived emotional intensity and BOLD responses in these regions during emotional video clips.

## Methods

### Participants

#### fMRI study

Forty-seven healthy participants aged between 20 and 30 years, fluent in English and with no history of neurological illness, took part in the study. Six participants were excluded from the analysis due to current or recurrent episodes of mental illness as assessed by the Mini International Neuropsychiatric Interview (Sheehan et al., 1998). Five further participants were excluded because of excessive head movement or poor task performance (<2 SD in empathic accuracy (EA) scores) and two participants had incomplete questionnaire data. The final dataset included 34 subjects (19 females, mean age: 24.0 years, SD: 2.7 years). The study received ethical approval from the Camberwell - St. Giles NHS Research Ethics Committee (14/LO/0477) and the University of Southampton Ethics Committee.

#### Normative data collection

To create a normative data set for the EAT and to validate the stimuli used in the fMRI task, an additional 73 healthy participants completed the EAT outside the MRI scanner. The same inclusion criteria as described above were applied. After excluding 13 participants due to current or recurrent episodes of mental illness, the final dataset included 60 healthy participants (36 females, mean age: 25.2 years, SD: 2.9 years). This aspect of the study was approved by the University of Southampton Ethics Committee.

### Tasks and stimuli

#### Video acquisition

Eleven native English-speaking students from the University of Southampton acted as targets (8 females, mean age: 20.1 years, SD: 1.64 years). Before filming they were asked to recall a specific autobiographical event (happy, sad, angry or frightened), in which they remembered feeling a strong emotion. Each target wrote a short summary of each event and rated its overall emotional intensity on a 9-point scale (from 1, ‘no emotion’ to 9, ‘very strong emotion’). For the emotional stimuli, only events with a rating of 5 or above were filmed. Each target provided one video clip for each emotion and one clip in which they described their bedroom (neutral condition). An adapted emotion elicitation strategy, which involved imagining being in the situation, was used before filming to reinstate the affective states the targets had felt during the events (Mantani et al., 2005). They were advised to refrain from making specific reference to their affective state (e.g. happy) but were allowed to use generic descriptions (e.g. upset) or descriptions of bodily symptoms (e.g. shaking). All targets were filmed from the shoulders upwards, in front of a black background, for standardisation purposes. Each clip lasted between 83 and 140 s (mean = 100.3, SD = 15.2). After filming each clip, targets watched the video and continuously rated their emotional intensity using the same 9-point scale as above. Ratings were made by using arrow keys on the keyboard to move a coloured square on the scale (this shifted by one point per button press). Starting point for all ratings was “1”.

For the fMRI study, the 6 video clips that were selected (one happy, one sad and one neutral video, featuring one female and one male target) were those which received high EA and target expressiveness scores in a pilot study with 13 participants (7 male, mean age: 21.54 years, SD: 2.37 years). A description of the target's gender, the emotional condition, the clip length and the target's rating of emotional intensity experienced during each clip is presented in Table 1. For pre-training and volume adjustment, one additional sad, one neutral and two happy clips were added (depicting different targets from the main experiment). For the data collection outside the MRI scanner, 27 expressive video clips were selected (7 happy clips, 7 sad clips, 3 angry clips, 3 frightened clips and 7 neutral clips) as well as two happy clips and one sad clip for pre-training purposes. The task and instructions for filming stimuli are available on request.

**Table 1:** Video clips displayed in order of presentation during the Empathic Accuracy Task with target's gender, emotional condition and length of the video clip and targets' average ratings of their own emotional intensity.

	video 1	video 2	video 3	video 4	video 5	video 6
target's gender	male	male	female	female	male	female
emotional condition	Happy (event)	Sad (event)	Neutral (control)	Happy (event)	Neutral (control)	Sad (event)
length [seconds]	89.96	89.44	90.48	84.76	84.24	104.52
average target's emotional intensity (SD)	5.44 (2.3)	6.25 (2.01)	2.19 (0.65)	7.44 (2.15)	1 (0)	7.57 (1.84)

#### Empathic accuracy task (EAT)

**Fig. 1:**
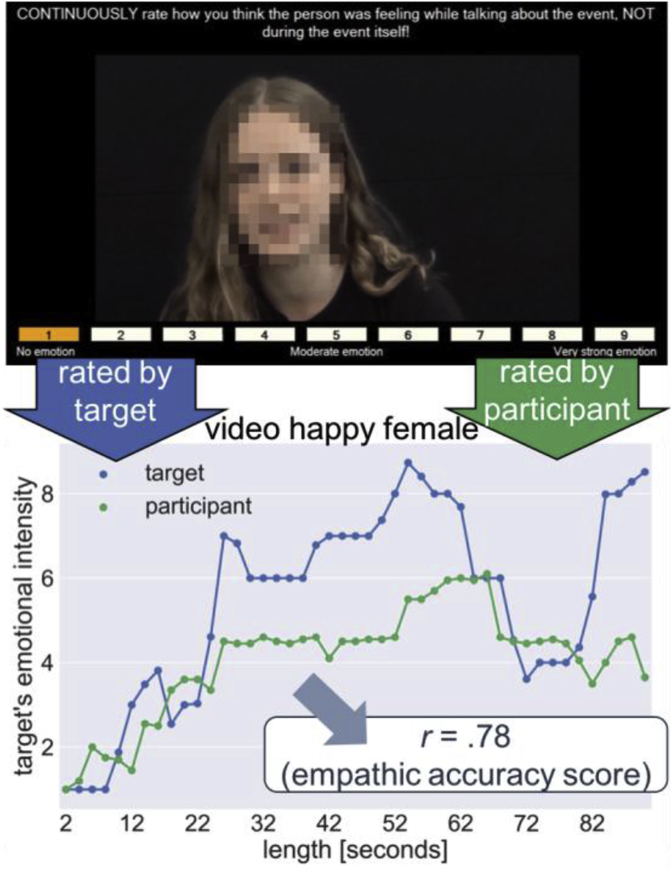
**Schematic representation of the Empathic Accuracy task and continuous rating scale data.** Top: example of a video clip and rating scale in the Empathic Accuracy Task. The target's identity has been disguised in this image. Bottom: Illustration of fluctuations in the *target's* emotional intensity, as rated by the target (blue) and an example participant's ratings (green). An Empathic Accuracy (EA) score was computed by correlating the participant's ratings and the target's ratings for each video clip.

Participants were instructed to continuously rate the perceived emotional intensity of the target (Fig. 1, top) using the same 9-point scale as above (from 1, ‘no emotion’ to 9, ‘very strong emotion’). In the fMRI study, participants used a button box to provide ratings. In the non-imaging study, participants used the computer's arrow keys. The default rating at the start of each video clip was no emotion (i.e. rating of 1). Following each clip, participants were asked: (1) which emotion the target felt most strongly (cognitive empathy: options of “happy”, “angry”, “surprised”, “sad”, “frightened” and “no emotion”); and (2) which emotion they themselves felt most strongly (i.e., affective empathy: same response options as above).

#### Interpersonal reactivity index

The Interpersonal Reactivity Index (IRI) is a widely-used self-report questionnaire that measures dispositional empathy using four subscales: fantasy (FS), empathic concern (EC), perspective taking (PT) and personal distress (PD; Davis, 1983).

#### Wechsler abbreviated scale of intelligence, Second Edition

The Wechsler Abbreviated Scale of Intelligence, Second Edition (WASI-II; Wechsler, 2011) is a widely-used and reliable test of general intelligence.

### Procedure

#### fMRI study

The EAT was part of the testing protocol of the English and Romanian Adoptees' Brain Imaging Study (for further details, see Sonuga-Barke et al., 2017). Participants gave written informed consent to participate in the study. All participants completed the MINI and WASI-II, and an online survey, which included the IRI. Participants received pre-training on the fMRI tasks prior to the scan, during which they were familiarised with the EAT and the scanning environment. After observing the experimenter demonstrating how to rate one happy clip, participants rated two clips (one sad, one happy) themselves, while lying in a mock scanner. In the actual EAT experiment, participants watched and rated the 6 video clips in a fixed order (Table 1). The task took approximately 12 min. Participants were reimbursed for around 6 h of their time with a £100 Amazon voucher.

#### Normative data collection

For the non-scanning study, participants gave written consent to participate. For pre-training, participants first watched the experimenter rate one happy clip before rating two practice video clips themselves. They then watched and rated 27 video clips in randomised presentation order, in a quiet testing room. This lasted approximately 40 min. Participants also completed an online survey, which included the IRI. Participants were reimbursed for their time with a £15 Amazon voucher.

### Behavioural data analysis

Participants' and targets' ratings were analysed using Matlab 8.2.0 (The MathWorks Inc., Natick, Massachusetts, United States) and SPSS (Version 22, IBM Corp., Armonk, New York, United States). All ratings were separated into 2 s bins and one time-weighted average rating was calculated for each bin. We then tested for correlations between the participants' and targets' ratings (Fig. 1, bottom). The resulting Pearson's correlation coefficient for each video clip and each participant is referred to as the EA score. As expected, the variance of the ratings was low for neutral clips. EA scores were therefore only calculated for emotional video clips. EA scores were then r-to-Z transformed to allow comparison between correlation coefficients (Fisher, 1915, 1921).

#### Behavioural analysis of fMRI sample

Paired t-tests examined whether Z-transformed EA scores, affective and cognitive empathy scores differed between happy and sad video clips. Moreover, paired t-tests were performed to test for differences in the average ratings of the target's emotional intensity between emotional and neutral as well as happy and sad video clips. A paired *t*-test was also used to test whether Z-EA scores differed between video clips that elicited “affect sharing” (participants reported feeling the same emotion as the target) compared to those that did not (participants reported a different emotion or no emotion). In addition, Pearson correlations were conducted to test for relationships between mean Z-EA scores, the IRI subscales and IQ.

#### Behavioural analysis of normative data sample

To examine whether the video clips presented in the fMRI study induced Z-EA scores comparable to those in the non-scanning sessions, two Pearson correlations were performed within the normative data sample. Considering happy and sad video clips separately, we examined the correlation between Z-EA scores based on the two video clips presented in the scanner and Z-EA scores based on all seven video clips from the respective emotional category. Moreover, intra-individual standard deviations were calculated based on (1) the four emotional video clips presented in the scanner and (2) all 20 emotional video clips. These were then compared with a paired *t*-test.

### fMRI data acquisition

Functional images were acquired on a General Electric MR750 3.0 T MR scanner with a 12-channel head coil. A T2*-weighted gradient echo, echo-planar imaging sequence was used, which covered 41 axial slices and recorded 347 vol acquired sequentially, descending (TR/TE 2000/30 ms, flip angle 75°, 64 × 64 matrix, 3 mm thick, field of view (FoV) = 247 mm). To facilitate fMRI data registration and normalisation, we also acquired a T1-weighted Magnetization Prepared Rapid Gradient Echo MPRAGE image (TR/TE 7312/3.02 ms, flip angle 11°, 256 × 256 matrix, 1.2 mm thick, 196 sagittal slices, FoV = 270 mm).

### fMRI data analysis

We used SPM12 for pre-processing and subject-level (first level) analyses (Wellcome Department of Cognitive Neurology, Institute for Neurology, London, UK). FSL was utilised for cerebrospinal fluid (CSF) regression and statistical nonparametric permutation inference at the group level (second level) with “randomise” (Winkler et al., 2014; FMRIB Analysis Research, Oxford Centre for Functional MRI of the Brain, Oxford, UK).

#### Preprocessing

After reorientation, the EPI files were first slice-time corrected (middle slice as reference). Images were then realigned to the first image and subsequently to the time series mean. The mean EPI image was co-registered to the T1-weighted image to allow for normalisation. The structural files were segmented and the resulting grey matter, white matter and CSF files were used to create a common group-specific template using group-wise DARTEL registration (Ashburner, 2007). This template was then employed to normalise the functional EPI files to MNI space. This step simultaneously resampled volumes (1.5 mm isotropic) and applied spatial smoothing (Gaussian FWHM kernel of 8 mm). Finally, for each participant, the time course signal of a CSF mask (top 5% from DARTEL CSF component) was extracted in native space.

#### Emotional vs neutral video clips

At the first level of analysis, each participant's pre-processed data were modelled as a block design using a general linear model framework. We included 3 separate regressors (happy, sad, neutral) encoding the predicted BOLD response associated with video presentation, formed by convolution of the canonical haemodynamic response function (HRF) with boxcars delimiting the video presentation.

We identified regional estimates of BOLD response associated with watching and rating the video clips. Separate parameter estimates for mean response during the emotional (happy and sad) and neutral category compared to the implicit baseline were produced. At the group level, in a random-effects model, paired t-tests were performed to identify clusters that were differentially activated when watching emotional video clips compared to neutral clips. Moreover, happy and sad clips were compared using paired t-tests.

#### Intra-individual variation in empathic accuracy

In accordance with Zaki and Ochsner (2009), Z-EA scores for each participant and each video clip were added as parametric modulators at the first level of analysis. On the group level, one sample t-tests were performed, to test whether the BOLD response during emotional video clips was modulated by intra-individual variations in Z-EA scores.

#### Correlation with emotional intensity ratings

We examined how the BOLD time series correlated with the participant's ratings of the target's emotional intensity. Scans were split and a model was fitted to each emotional video clip in turn. The continuous ratings of the target's emotional intensity for each 2 s bin as rated by the participant were entered as regressors of interest. At the group level, one-sample t-tests assessed whether the relationship between BOLD response and changes in the emotional intensity ratings was significantly observed in any brain region across the group.

#### Exploratory analysis: impact of affect sharing

To examine differences in BOLD response for video clips that induced affect sharing compared to those that did not, we conducted an exploratory post-hoc analysis. We included the 20 participants who showed affect sharing in response to some, but not all video clips in order to be able to create 3 separate conditions in the first level in a block design (shared, non-shared, neutral). For each participant, emotional videos that induced affect sharing (participants reported to have the same emotion as the target) were included in the shared condition, while emotional videos that did not elicit affect sharing (participants reported to have a different emotion than the target or no emotion) were modelled in the non-shared condition. Separate parameter estimates for mean response during affect shared, non-shared and neutral video clip presentation compared to the implicit baseline were calculated. At the group level, paired t-tests were performed to identify clusters that were differentially activated when watching video clips that induced affect sharing compared to non-shared clips.

#### Movement, scanner drifts and multiple comparisons correction

As well as the regressors described above, all analyses included seven movement parameters (six standard parameters as well as volume-to-volume movement) as nuisance regressors. For each volume-to-volume movement exceeding 1 mm, an additional regressor was included marking the location of that volume and those immediately adjacent (for a summary of volume-to-volume movement see Supplementary Table 1). The CSF regressor was also included as a nuisance regressor. To control for task-related hand movement artefacts, button presses were included as condition of no interest. To investigate the effect of controlling for button presses, we additionally repeated all analyses without including this condition. Moreover, we compared button presses during emotional video clips with button presses during neutral video clips as separate conditions to ensure that activity relating to emotion processing was not partialled out.

Data were high pass filtered with a threshold of 209 s, which corresponds to twice the length of the longest video clip, to control for scanner drifts.

Results reported are based on Family-Wise Error (FWE) corrected threshold-free cluster enhancement (TFCE: *p*_FWE_ < 0.05 (Smith and Nichols, 2009)). For each significant cluster, the peak activations with a minimum inter-peak distance of 20 voxels are reported to account for the wide-spanning clusters found in our analyses.

## Results

### Behavioural data

#### Behavioural analysis of the fMRI sample

On average, participants had high EA scores (mean *r* = .75, mean intra-individual standard deviation (iSD) = .35, range = .13 to .97). Fisher's Z-transformed (Z-)EA scores were slightly, but significantly, lower for sad video clips (mean Z-EA = 0.97, SD = 0.21) than happy ones (mean Z-EA = 1.16, SD = 0.19; *t* (33) = 5.17, *p* < .001). As expected, participants' average ratings of the target's emotional intensity were higher for emotional than for neutral video clips (mean emotional = 5.18, mean neutral = 1.75, *t* (33) = 15.29, *p* < .001), with higher ratings for sad compared to happy ones (mean sad = 5.49, mean happy = 4.87, *t* (33) = 3.02, *p* < .01).

On average, participants correctly inferred the target's emotion in 90.4% of clips (emotion identification, SD = 15.1%), with no difference between happy and sad clips (*t* (33) = −0.33, *p* = .74). They also reported experiencing the same emotion as the target for the majority of the emotional video clips (affect sharing, mean = 72.8%, SD = 28.5%), with a higher degree concordance for sad (mean = 79.4%, SD = 32.8%) compared to happy clips (mean = 66.2%, SD = 31.9%; *t* (33) = 2.5, *p* < .05). 13 participants shared the target's emotion in every emotional video clip while one participant did not show affect sharing in any of the clips. For the remaining 20 participants who showed a mix of affect sharing and non-sharing, Z-EA scores did not differ for videos that elicited affect sharing (mean Z-EA = 1.09, SD = .24) compared to those that did not (mean Z-EA = 1.06, SD = .33, *t* (19) = .36, *p* = .72).

Additionally, we found a positive correlation between participants' mean Z-EA scores and IRI perspective-taking (*r* = .48, *p* < .01). No significant correlations were found between mean Z-EA scores and the other IRI subscales or estimated IQ (all *ps* > .09).

Note that while we used Pearson's product-moment correlation, alternative methods for assessing agreement are available such as the intraclass correlation coefficient. EA scores derived using this measure were highly correlated (r = 0.89) with Pearson's correlations. We chose the latter for two reasons. First, we were able to confirm our findings after partialling out dependency over time of the ratings (data not shown) and second, we wished to maintain compatibility with previous studies using similar tasks that also based estimates of inter-rater agreement on Pearson's correlations.

#### Behavioural analysis of the normative data sample

The analysis showed that the mean Z-EA scores for the video clips presented in the fMRI study were strongly positively correlated with Z-EA scores for the seven clips presented in the normative data study (happy: *r* = .82, *p* < .001; sad: *r* = .77, *p* < .001). Furthermore, the intra-individual standard deviation of the four emotional video clips presented in the scanner (mean iSD = .36) did not differ from the individual standard deviation across all 20 emotional video clips presented outside the scanner (mean iSD = .39, *t* (59) = −1.64, *p* = .11).

### fMRI data

#### Emotional vs. neutral video clips

**Fig. 2:**
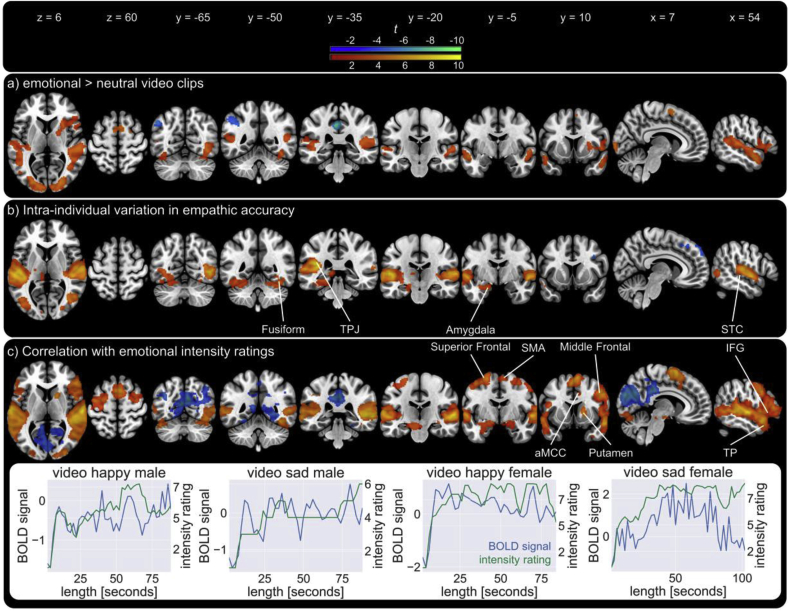
**Neural substrates of changes in empathy.** a) Significant brain activations when viewing emotional video clips compared to neutral ones. b) Regions significantly positively (red) and negatively (blue) modulated by variations in empathic accuracy (Z-EA scores). c) top: Brain areas significantly positively correlated over time with the participants' ratings of the target's emotional intensity. bottom: BOLD response (after first level regression) of significant clusters (blue) and participant's ratings of the target's emotional intensity (green) of one exemplary participant. Key: STC - superior temporal cortex, TP - temporal pole, TPJ - temporoparietal junction, IFG - inferior frontal gyrus, SMA - supplementary motor area, aMCC - anterior midcingulate cortex.

Group-level analysis revealed a higher BOLD response during emotional compared to neutral clips in a large cluster spanning multiple regions, with peak activations in bilateral occipital poles and inferior lateral occipital cortex (Fig. 2a, Table 2). The cluster included bilateral posterior and anterior superior temporal cortex (STC), as well as bilateral temporal pole (TP), bilateral planum temporale and bilateral posterior temporoparietal junction (pTPJ). Higher activation was also seen in right inferior frontal gyrus (IFG; including pars triangularis and opercularis), with the cluster extending into right anterior insular cortex (AI) and right putamen. A second cluster showed higher activation in supplementary motor area (SMA). While participants were watching neutral compared to emotional video clips, activation was higher in left superior lateral occipital cortex, left posterior cingulate cortex (PCC) and left precuneus. Significant activation was similar, albeit more widespread, when not controlling for button presses (see Supplementary Fig. 1 and Supplementary Tables 2–4). Moreover, when analysing the button press condition separately for emotional and neutral video clips, no brain regions showed significant differences between both button press conditions.

**Table 2:** Significant clusters and their peak activations for the contrasts emotional > neutral video clips and neutral > emotional video clips (threshold-free cluster enhancement *p*_FWE_ < 0.05).

Cluster	Anatomical region	Hemisphere	Cluster size	MNI coordinates [mm]			Peak-level *t*
				x	y	z	
**emotional > neutral video clips**							
1	Occipital Pole	R	9901	20	−94	−2	7.33
	Inferior Lateral Occipital Cortex	L		−32	−90	−6	6.86
	Anterior Superior Temporal Cortex	R		52	2	−18	6.21
	Occipital Pole	L		−16	−94	12	5.94
	Inferior Lateral Occipital Cortex	R		40	−70	−4	5.1
	Posterior Superior Temporal Cortex	R		56	−28	6	4.57
	Insular Cortex	R		28	16	8	4.39
	Temporal Occipital Fusiform Gyrus	R		40	−50	−16	4.15
	Frontal Operculum Cortex	R		48	16	0	4.02
	Occipital Fusiform Cortex	L		−36	−70	−16	4.01
	Occipital Pole	L		−10	−100	−14	3.44
2	Anterior Superior Temporal Cortex	L	2243	−52	−6	−14	5.32
	Posterior Supramarginal Cortex	L		−56	−44	14	4.45
	Middle Temporal Gyrus	L		−44	−32	−2	3.85
	Planum Temporale	L		−60	−20	6	3.48
3	Supplementary Motor Cortex	R	250	6	4	60	5.04
4	Temporal Pole	L	6	−46	18	−26	3.53
**neutral > emotional video clips**							
1	Superior Lateral Occipital Cortex	L	1014	−34	−80	40	6.59
	Superior Lateral Occipital Cortex	L		−44	−84	22	5.86
2	Posterior Cingulate Gyrus	L	207	−4	−38	40	8.99
3	Precuneus Cortex	L	86	−14	−60	14	6.2
4	Superior Lateral Occipital Cortex	R	54	36	−76	42	5.32
5	Lingual Gyrus	R	2	34	−38	−10	5.49
6	Planum Temporale	R	2	30	−30	−20	5.46

To explore differences between the different emotion conditions, we also directly compared happy and sad video clips. Activation in the bilateral STC was higher during happy compared to sad clips, while the right paracingulate gyrus and right precuneus showed higher activation during sad video clips (see Supplementary Fig. 2 and Supplementary Table 5).

#### Intra-individual variation in empathic accuracy

Participants' intra-individual variations in Z-EA scores were positively related to activation in clusters spanning the bilateral STC, planum temporale, TP and pTPJ, left hippocampus and left amygdala. Activity in the bilateral inferior lateral occipital cortex and fusiform cortex was also positively related to Z-EA scores (Fig. 2b, Table 3). Activation in the bilateral paracingulate gyrus and right frontal pole as well as the right middle frontal gyrus was significantly negatively modulated by Z-EA scores.

**Table 3:** Significant clusters and their peak activations for the modulation of BOLD-response by intra-individual variation of Z-EA scores (threshold-free cluster enhancement *p*_FWE_ < 0.05).

Cluster	Anatomical region	Hemisphere	Cluster size	MNI coordinates [mm]			Peak-level *t*
				x	y	z	
**Positively related to Z-EA scores**							
1	Posterior Superior Temporal Cortex	L	9036	−62	−26	10	9.88
	Planum Temporale	L		−38	−34	14	9.17
	Temporal Pole	L		−54	−2	−2	7.02
	Hippocampus	L		−20	−14	−20	6.50
	Inferior Lateral Occipital Cortex	L		−44	−72	4	6.03
	Posterior Temporal Fusiform Cortex	L		−38	−42	−26	4.67
	Occipital Fusiform Cortex	L		−20	−90	−18	4.61
2	Planum Temporale	R	2421	64	−16	8	7.37
	Planum Temporale	R		34	−28	14	4.93
3	Inferior Lateral Occipital Cortex	R	2315	46	−66	0	7.80
	Occipital Fusiform Cortex	R		22	−88	−8	5.37
**Negatively related to Z-EA scores**							
1	Paracingulate Gyrus	R	275	2	22	48	4.11
	Frontal Pole	R		10	62	36	4.01
	Paracingulate Gyrus	L		−6	44	30	3.81
2	Middle Frontal Gyrus	R	31	36	14	32	4.42

#### Correlation with emotional intensity ratings

**Fig. 3:**
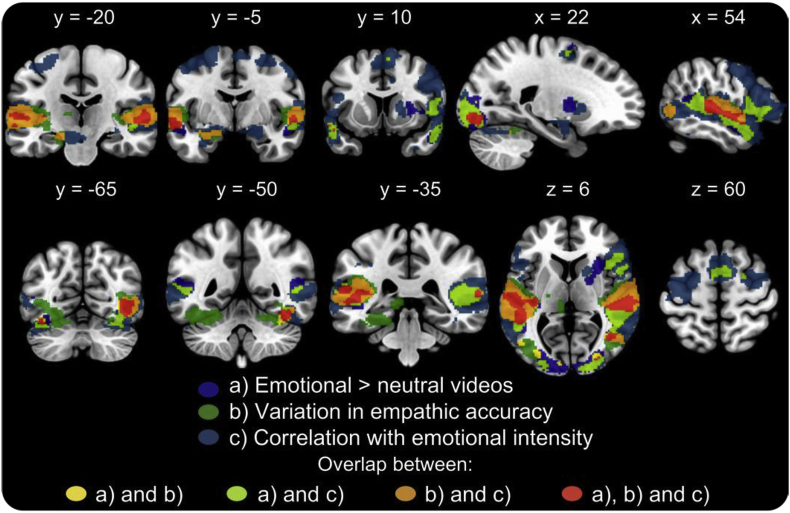
Binarised overlay of activations related to a) emotional compared to neutral video clips, b) variation positively related to empathic accuracy and c) positive correlation with emotional intensity.

While watching emotional video clips, participants' fluctuations in ratings of the targets' emotional intensity were positively correlated over time with changes in BOLD response in multiple brain regions (Fig. 2c, Table 4). Associations were found in multiple clusters including bilateral posterior STC, bilateral TP, bilateral IFG (including pars triangularis and opercularis), bilateral SMA, bilateral middle and superior frontal cortices, right anterior midcingulate cortex (aMCC), right AI, bilateral amygdala, bilateral putamen as well as pTPJ and right temporal occipital and anterior temporal fusiform cortex. Emotional intensity ratings and BOLD-response were negatively correlated in the in the bilateral superior lateral occipital cortex, PCC, and precuneus. Fig. 3 shows a binarised overlay of significant clusters in the different analyses.

**Table 4:** Significant clusters and their peak activations for the correlation between BOLD-response and the participants' ratings of the target's emotional intensity (threshold-free cluster enhancement *p*_FWE_ < 0.05).

Cluster	Anatomical region	Hemisphere	Cluster size	MNI coordinates [mm]			Peak-level *t*
				x	y	z	
**Positive correlation with participants' emotional intensity ratings**							
1	Posterior Superior Temporal Cortex	R	24492	58	−16	0	9.56
	Posterior Middle Frontal Cortex	R		62	−36	0	8.26
	Temporal Pole	R		58	8	−16	8.19
	Planum Temporale	L		−64	−14	6	8.05
	Putamen	R		26	−92	−6	7.4
	Temporal Pole	L		−56	4	−10	7.04
	Middle Frontal Gyrus	R		48	8	38	6.92
	Middle Temporal Gyrus, temporooccipital part	R		46	−56	2	6.49
	Temporal Occipital Fusiform Gyrus	R		40	−46	−16	6.06
	Temporal Occipital Fusiform Gyrus	L		−46	−64	−28	5.7
	Insular Cortex	R		38	2	−20	5.49
	Middle Temporal Gyrus, temporooccipital part	L		−64	−44	6	5.31
	Inferior Frontal Gyrus, pars triangularis	R		56	28	8	5.04
	Temporal Pole	L		−44	20	−26	4.93
	Occipital Fusiform Gyrus	L		−30	−82	−18	4.87
	Planum Temporale	L		−40	−36	10	4.84
	Amygdala	L		−18	−6	−14	4.78
2	Supplementary Motor Cortex	R	1735	6	8	66	6.85
	Anterior Midcingulate Gyrus	R		8	14	38	3.74
3	Precentral Gyrus	L	1714	−40	−8	56	5.64
	Superior Frontal Gyrus	L		−24	2	72	4.96
4	Postcentral Gyrus	L	206	−48	−26	40	4.05
5	Putamen	R	173	18	10	6	4.67
**Negative correlation with participants' emotional intensity ratings**							
1	Cuneus Cortex	R	10193	10	−86	24	6.42
	Posterior Cingulate Cortex	R		2	−34	38	5.48
	Precuneus Cortex	L		−4	−66	16	5.3
	Superior Lateral Occipital Cortex	R		38	−74	22	5.25
	Precuneus Cortex	L		−12	−58	34	4.82
	Lingual Gyrus	R		26	−52	−4	4.66
	Superior Lateral Occipital Cortex	R		44	−64	46	3.99
2	Superior Lateral Occipital Cortex	L	466	−38	−70	32	4.69
3	Temporal Occipital Fusiform Cortex	L	121	−24	−56	−12	3.23
4	Superior Lateral Occipital Cortex	L	32	−50	−74	26	3.77

#### Exploratory analysis: impact of affect sharing

For emotional video clips, there were no significant differences in BOLD response between clips that elicited, versus those that did not elicit, affect sharing (i.e. participants reported experiencing the same emotion as the target after providing their continuous ratings).

## Discussion

We used a modified version of the EAT to study neural substrates of empathic accuracy and to gain a better understanding of its underlying components. We demonstrated that fluctuations in participants' perceived emotional intensity ratings are correlated with activation in a network of brain regions previously implicated in empathy and broader aspects of social cognition (i.e., mentalising). More specifically, consistent with our first hypothesis, we observed increased activation in brain regions associated with empathy and mentalising when participants watched emotional compared to neutral clips. Supporting our second hypothesis, we found a positive correlation between intra-individual variations in empathic accuracy and the temporal lobe, “mentalising” regions of the same network. Confirming our third hypothesis, we found a correlation between fluctuations in ratings of the targets' perceived emotional intensity over time and activity in these same regions. This network of brain regions appears not only to have a general role in emotion and empathic processing but is also sensitive to *variations* in the intensity of others' emotions.

The superior temporal sulcus (STS), temporoparietal junction (TPJ), and temporal pole (TP) have consistently been associated with mentalising (Molenberghs et al., 2016). In our study, these areas were more active with higher EA, i.e. when participants were more accurate at tracking the target's emotion. Beyond this, we could also show these regions are sensitive to fluctuations in perceived emotional intensity of others. The STS is thought to facilitate mentalising by interpreting social aspects of observed biological motion (Allison et al., 2000, Molenberghs et al., 2016) and the region has been implicated in EA (Zaki et al., 2009, Kral et al., 2017). The TPJ is involved in inferring other people's temporary mental states (Van Overwalle and Baetens, 2009) while the TP's role in mentalising is thought to involve the integration of multimodal information and recollection of social scripts (Greven and Ramsey, 2017; Frith, 2007). Combined, these brain regions are involved in distinct emotional and cognitive processes that are required to perform our modified EAT: they are integral for the successful tracking of others' emotional intensity and correlate positively with intra-individual variations in EA.

The anterior insula (AI), anterior midcingulate cortex (aMCC), inferior frontal gyrus (IFG) and supplementary motor area (SMA) have previously been implicated in empathy tasks and are associated with the affect sharing component of empathy (or affective empathy) (Fan et al., 2011). Together these regions are implicated in the emotional processing of the modified EAT stimuli. Most importantly, we could show for the first time that their activity tracks the perceived emotional intensity of others. However, activity in these brain regions was not sensitive to changes in EA and thus seems more tied to the subjective perception of other's feelings.

This suggests it is the time-series variation in activation in the temporal lobe regions (STS, TPJ, TP) that might be informative for accurately tracking other people's emotions, while activation in the frontal regions (AI, ACC, IFG, SMA) represents a different emotion processing component that does not vary with changes in EA (Fig. 3). This is consistent with previous studies on EA, which showed either no correlation between EA and activity in the above frontal regions (Zaki et al., 2009) or, in the case of adolescents, a negative correlation between EA and ACC and AI activation (Kral et al., 2017). Furthermore, we could not replicate an association between EA and activity in the inferior parietal lobe, a region implicated in motor imitation and previously interpreted as an affective processing component of EA (Zaki et al., 2009). Taken together, these findings provide evidence that EA is more closely related to the concept of cognitive empathy and mentalising than affective empathy and emotion sharing. The role of EA in cognitive but not affective empathy is further supported by the positive correlation between EA scores and the perspective-taking scale of a well-established self-report measure of empathy (the IRI) but not with other more affective subscales such as empathic concern. Moreover, participants' average EA scores did not differ between videos where they shared the same emotion as the target compared to those were they did not, which again suggests that emotion sharing is neither necessary for, nor facilitates, EA.

Even if EA does only relate to cognitive but not affective empathy, the EAT as a task successfully elicited affective empathy in most of our participants – they reported sharing the target's emotion in 73% of the emotional video clips. However, there were no significant differences in brain activity when rating videos where participants shared the same emotion compared to videos where they did not. This further supports our hypothesis that the higher activation in aMCC, AI, SMA and IFG during emotional clips is associated with more basal, empathy-independent aspects of emotion processing.

Higher activation of the bilateral STS could also be seen during happy compared to sad video clips, while the right paracingulate gyrus was more highly activated during sad video clips. This is in line with our behavioural findings of, on average, higher EA scores during happy video clips, which suggest more successful tracking and mentalising of the target's emotion, while sad video clips induced higher rates of affect sharing among participants. The paracingulate gyrus has previously been implicated in affective empathy (Fan et al., 2011).

During the modified EAT, participants rated fluctuations in emotional intensity rather than valence as this allowed a comparable rating scale across different distinct emotions. Furthermore, previous literature suggests distinct neural correlates for processing emotional intensity and valence (Lewis et al., 2006), with the amygdala being associated with intensity and the orbitofrontal cortex with valence. In agreement with this, we found that bilateral activation of amygdala but not the orbitofrontal cortex covaried with the emotional intensity of the targets. Unexpectedly activation in the precuneus – a region implicated in self-referential processing (Northoff et al., 2006) – was stronger during neutral versus emotional clips and correlated negatively with emotional intensity ratings. The precuneus is associated with visual-spatial imagery (Cavanna and Trimble, 2006, Spreng and Grady, 2010) and is a component of the default mode network (Laird et al., 2009). Higher activation during the neutral videos in which participants described their bedroom, might be explained by higher visual-spatial imagery and an increased tendency for mind-wandering during these less engaging parts of the task (Smallwood et al., 2016).

Empathy is a complex and dynamic process, which requires multiple higher order functions (Tousignant et al., 2017) such as emotion recognition, multimodal sensory integration, self-other distinction and continuous processing of valence and intensity information. Compared to other commonly used empathy tasks, the modified EAT used a more naturalistic setting to examine which brain regions track fluctuations over time in perceived emotional intensity of others and intra-individual variations in empathic accuracy. Previous studies in the empathy and mentalising literature have largely focused on simplistic stimuli (e.g. static images of hands in painful situations). Compared to these earlier studies, we found that regions that have been separately implicated in mentalising and empathy were all involved in performing the modified EAT. However, only brain regions previously associated with mentalising were found to covary with EA, while regions previously implicated in classic affective empathy paradigms were positively correlated with the emotional intensity of others but were not sensitive to changes in EA. In this more naturalistic and complex task, it seems that an interplay between brain networks associated with mentalising and empathy enables the accurate tracking of other's emotions. Furthermore, these regions were sensitive to fluctuations in perceived emotional intensity of others, which serves as a potential mechanism for successful communication between these networks to achieve empathic accuracy.

A possible limitation of our study was the lower number of emotional video clips in comparison to previous studies on EA (Zaki et al., 2009, Kral et al., 2017). This study was conducted within the framework of a larger project, and thus the scanning time was limited. However, we showed that our chosen video clips led to very similar EA scores relative to those obtained with the larger dataset of 27 video clips in the norm sample. More importantly, the intra-individual variation across videos was also comparable to that seen for the full set of video clips.

The study had a number of strengths. The original EAT (Zaki et al., 2009) represented an important advance in empathy research, as it was the first task to utilise naturalistic stimuli and assess EA in an fMRI context. In this modified EAT, the stimuli used for fMRI purposes had been validated in a separate behavioural study. Moreover, we added a neutral control condition, which allowed us to identify brain regions that are generally more active during emotional video clips irrespective of empathic accuracy. Future studies could employ this paradigm to study psychiatric populations with empathy deficits (e.g., adolescents with Conduct Disorder; Martin-Key et al., 2017). By additionally taking the neutral control condition into account, one could examine whether emotional clips were ‘neutral-like’ in those with low EA scores. For future studies, it would be worth considering incorporating neutral videos with varying topics other than bedroom descriptions to ensure continued engagement throughout the task (see Kanske et al., 2015 for possible examples). Furthermore, we introduced the measurement of emotional intensity rather than valence, which is more closely related to the concept of empathy. This also made the video clips of different emotions comparable and allowed a more fine-grained analysis of changes over time in activation related to the emotional intensity of others. Together, we propose that the three analysis techniques used in this study, should be employed in conjunction to allow a comprehensive study of empathic accuracy and its different components.

In conclusion, we provide the first evidence that the modified EAT is a suitable paradigm for studying empathy and its underlying components. We show that, while the modified EAT successfully induces both affective and cognitive empathy, EA relies more on cognitive empathy than affect sharing. The neutral control condition and the valence-independent rating scale represent valuable additions to the task. The fMRI data analysis techniques developed and described here may prove valuable in characterising differences between healthy participants and participants with psychiatric conditions associated with empathy deficits.

## Funding

This work was funded by a project grant from the Medical Research Council to ESB, MM and GF (MR/K022474/1).

## Declaration of interest

We do not have any financial, institutional or other relationships that might lead to a conflict of interest.
