# Supplementary material for: Tracking emotions in the brain – Revisiting the Empathic Accuracy Task
Source: Neuroimage. 2018 Sep;178:677–86. doi: 10.1016/j.neuroimage.2018.05.080 (PMC6057276; doi:10.1016/j.neuroimage.2018.05.080)
Supplement: Supplementary Information [file mmc1.docx]

# Tracking emotions in the brain – Revisiting the Empathic Accuracy Task

# Supplementary Material

## Volume-to-volume movement summary

Supplementary Table 1: Volume-to-volume movement [in mm] for each emotional condition (happy, sad, neutral).

| Video condition | Average volume-to-volume movement (SD) [mm] | Percentage of volumes exceeding a movement of 1 mm |
| --- | --- | --- |
| Happy | 0.05 (0.03) | 0.14% |
| Sad | 0.05 (0.02) | 0.12% |
| Neutral | 0.06 (0.02) | 0.03% |

On average, there was no significant difference between happy, sad and neutral video clips in volume-to-volume movement (*F(2,66)* = 0.729, *p = .45*).

## Results without controlling for button presses


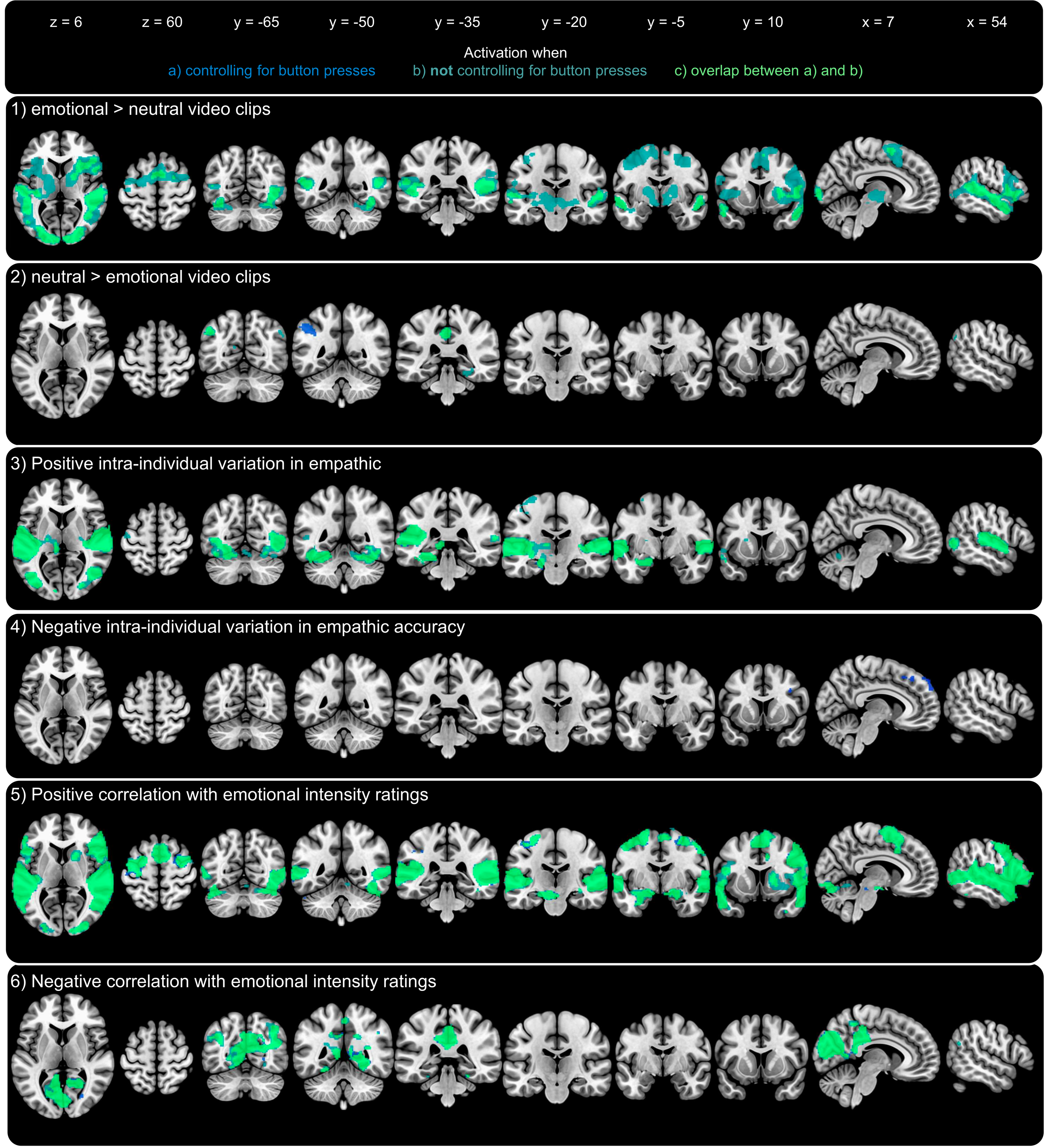


Supplementary Figure 1: Differential results when a) controlling for button presses (dark blue), b) not controlling for button presses (cyan) and c) the overlap between both analyses (bright green).

### Emotional vs neutral video clips

Supplementary Table 2: Significant clusters and their peak activations for the contrasts emotional > neutral video clips and neutral > emotional video clips when not controlling for button presses (threshold-free cluster enhancement *p*_FWE_ < 0.05). Brain regions highlighted with asterisks were not engaged with button presses included.

| Cluster | Anatomical region | Hemisphere | Cluster size | MNI coordinates [mm] | | | Peak-level *t* |
| --- | --- | --- | --- | --- | --- | --- | --- |
|  |  |  |  | x | y | z |  |
| **emotional > neutral video clips** | | | | | | | |
| 1 | Inferior Lateral Occipital Cortex | L | 30679 | -32 | -88 | -6 | 7.37 |
|  | Occipital Pole | R |  | 20 | -94 | -2 | 7.28 |
|  | Frontal Operculum Cortex | R |  | 46 | 16 | -2 | 7 |
|  | Anterior Superior Temporal Cortex | R |  | 52 | 2 | -18 | 6.89 |
|  | Supplementary Motor Cortex | R |  | 4 | 6 | 60 | 6.58 |
|  | Inferior Lateral Occipital Cortex | R |  | 42 | -68 | -4 | 6.11 |
|  | Occipital Pole | L |  | -16 | -94 | 12 | 5.86 |
|  | Insular Cortex | R |  | 28 | 14 | 8 | 5.42 |
|  | Superior Frontal Gyrus* | R |  | 24 | -2 | 56 | 5.41 |
|  | Precentral Gyrus* | R |  | 48 | 2 | 42 | 5.33 |
|  | Posterior Superior Temporal Gyrus | L |  | -52 | 2 | -18 | 5.08 |
|  | Posterior Supramarginal Cortex | R |  | 52 | -40 | 14 | 4.85 |
|  | Cerebellum* | R |  | 34 | -54 | -26 | 4.79 |
|  | Posterior Supramarginal Cortex | L |  | -56 | -44 | 14 | 4.78 |
|  | Precentral Gyrus* | L |  | -48 | -2 | 40 | 4.71 |
|  | Occipital Fusiform Cortex | L |  | -38 | -68 | -16 | 4.46 |
| **neutral > emotional video clips** | | | | | | | |
| 1 | Superior Lateral Occipital Cortex | L | 769 | -34 | -80 | 40 | 7.66 |
| 2 | Posterior Cingulate Gyrus | L | 294 | -4 | -38 | 40 | 9.67 |
| 3 | Precuneus Cortex | L | 160 | -14 | -60 | 14 | 6.53 |
| 4 | Planum Temporale | R | 146 | 30 | -30 | -20 | 6.17 |
| 5 | Superior Lateral Occipital Cortex | R | 100 | 36 | -76 | 42 | 5.39 |
| 6 | Posterior Temporal Fusiform Cortex* | L | 47 | -24 | -42 | -16 | 6.76 |
| 7 | Precuneus Cortex* | R | 8 | 12 | -56 | 14 | 5.69 |

Intra-individual variation in empathic accuracy

Supplementary Table 3: Significant clusters and their peak activations for the modulation of BOLD-response by intra-individual variation of Z-EA scores when not controlling for button presses (threshold-free cluster enhancement *p*_FWE_ < 0.05). Brain regions highlighted with asterisks were not engaged with button presses included.

| Cluster | Anatomical region | Hemisphere | Cluster size | MNI coordinates [mm] | | | Peak-level *t* |
| --- | --- | --- | --- | --- | --- | --- | --- |
|  |  |  |  | x | y | z |  |
| **Positively related to Z-EA scores** | | | | | | | |
| 1 | Posterior Superior Temporal Cortex | L | 10626 | -62 | -26 | 10 | 9.88 |
|  | Planum Temporale | L |  | -38 | -34 | 14 | 9.37 |
|  | Temporal Pole | L |  | -54 | 0 | -2 | 7.29 |
|  | Inferior Lateral Occipital Cortex | L |  | -44 | -72 | 4 | 6.28 |
|  | Hippocampus | L |  | -18 | -14 | -18 | 5.95 |
|  | Posterior Temporal Fusiform Cortex | L |  | -38 | -42 | -28 | 4.67 |
|  | Occipital Fusiform Cortex | L |  | -20 | -88 | -18 | 4.63 |
| 2 | Inferior Lateral Occipital Cortex | R | 3758 | 48 | -68 | 0 | 7.8 |
|  | Occipital Fusiform Cortex | R |  | 24 | -88 | -8 | 5.45 |
| 3 | Planum Temporale | R | 2716 | 64 | -18 | 8 | 7.58 |
|  | Planum Temporale | R |  | 34 | -28 | 14 | 4.9 |
| 4 | Precentral Gyrus* | L | 440 | -26 | -10 | 66 | 4.72 |
|  | Postcentral Gyrus* | L |  | -44 | -28 | 44 | 4.22 |
| **Negatively related to Z-EA scores** | | | | | | | |
|  | no significant clusters | |  |  |  |  |  |

Correlation with emotional intensity ratings

Supplementary Table 4: Significant clusters and their peak activations for the correlation between BOLD-response and the participants’ ratings of the target’s emotional intensity when not controlling for button presses (threshold-free cluster enhancement *p*_FWE_ < 0.05). Brain regions highlighted with asterisks were not engaged with button presses included.

| Cluster | Anatomical region | Hemisphere | Cluster size | MNI coordinates [mm] | | | Peak-level *t* |
| --- | --- | --- | --- | --- | --- | --- | --- |
|  |  |  |  | x | y | z |  |
| **Positive correlation with participants' emotional intensity ratings** | | | | | | | |
| 1 | Posterior Superior Temporal Cortex | R | 30113 | 58 | -14 | 0 | 9.66 |
|  | Posterior Middle Frontal Cortex | R |  | 62 | -36 | 0 | 8.33 |
|  | Temporal Pole | R |  | 58 | 6 | -16 | 8 |
|  | Planum Temporale | L |  | -64 | -14 | 6 | 7.9 |
|  | Putamen | R |  | 26 | -90 | -4 | 7.89 |
|  | Supplementary Motor Cortex | R |  | 6 | 8 | 66 | 7.54 |
|  | Middle Frontal Gyrus | R |  | 50 | 8 | 36 | 7.42 |
|  | Middle Temporal Gyrus,  temporooccipital part | R |  | 46 | -56 | 2 | 6.83 |
|  | Posterior Middle Temporal Cortex* | L |  | -66 | -32 | -4 | 6.59 |
|  | Temporal Pole | L |  | -56 | 4 | -12 | 6.56 |
|  | Precentral Gyrus | L |  | -40 | -8 | 56 | 6.16 |
|  | Insular Cortex | R |  | 38 | 2 | -20 | 6.11 |
|  | Temporal Occipital Fusiform Gyrus | L |  | -46 | -64 | -28 | 6.09 |
|  | Temporal Occipital Fusiform Gyrus | R |  | 40 | -48 | -16 | 5.91 |
|  | Middle Temporal Gyrus,  temporooccipital part | L |  | -54 | -50 | 2 | 5.57 |
|  | Planum Temporale | L |  | -40 | -36 | 10 | 5.2 |
|  | Superior Frontal Gyrus | L |  | -22 | -2 | 70 | 5.15 |
|  | Temporal Pole | L |  | -42 | 20 | -26 | 5.14 |
|  | Inferior Frontal Gyrus, pars triangularis | R |  | 50 | 34 | 6 | 5.12 |
| 2 | Putamen | R | 713 | 18 | 10 | 6 | 4.56 |
| **Negative correlation with participants' emotional intensity ratings** | | | | | | | |
| 1 | Cuneus Cortex | R | 12731 | 10 | -86 | 24 | 6.77 |
|  | Posterior Cingulate Cortex | R |  | 2 | -34 | 38 | 5.78 |
|  | Precuneus Cortex | R |  | 8 | -62 | 22 | 5.5 |
|  | Superior Lateral Occipital Cortex | R |  | 38 | -74 | 22 | 4.98 |
|  | Precuneus Cortex | L |  | -12 | -58 | 32 | 4.85 |
|  | Temporal Occipital Fusiform Gyrus | R |  | 26 | -54 | -10 | 4.82 |
|  | Superior Lateral Occipital Cortex | L |  | -36 | -74 | 32 | 4.65 |
| 2 | Frontal Pole* | R | 39 | 0 | 60 | 2 | 5.35 |

## Happy vs sad video clips

Supplementary Table 5: Significant clusters and their peak activations for the contrasts happy > sad video clips and sad > happy video clips (threshold-free cluster enhancement *p*_FWE_ < 0.05).

| Cluster | Anatomical region | Hemisphere | Cluster size | MNI coordinates [mm] | | | Peak-level *t* |
| --- | --- | --- | --- | --- | --- | --- | --- |
|  |  |  |  | x | y | z |  |
| **happy > sad video clips** | | | | | | | |
| 1 | Heschl's Gyrus | L | 2709 | -46 | -26 | 8 | 9.95 |
|  | Anterior Superior Temporal Gyrus | L |  | -60 | -8 | 0 | 9.72 |
|  | Parietal Operculum Cortex | L |  | -48 | -40 | 26 | 4.81 |
| 2 | Heschl's Gyrus | R | 2494 | 44 | -24 | 10 | 11.8 |
|  | Anterior Superior Temporal Gyrus | R |  | 62 | -2 | -2 | 10.1 |
| 2 | Posterior Superior Temporal Gyrus | R |  | 70 | -34 | 6 | 4.68 |
| 3 | Occipital Fusiform Gyrus | R | 87 | 38 | -64 | -20 | 5.5 |
| **sad > happy video clips** | | | | | | | |
| 1 | Superior Frontal Gyrus | R | 789 | 10 | 32 | 48 | 6 |
|  | Paracingulate Gyrus | R |  | 6 | 46 | 26 | 5.63 |
| 2 | Angular Gyrus | R | 261 | 56 | -54 | 28 | 5.56 |
| 3 | Posterior Cingulate Gyrus | R | 196 | 6 | -32 | 38 | 4.98 |
| 4 | Precuneus Cortex | R |  | 8 | -54 | 38 | 4.4 |


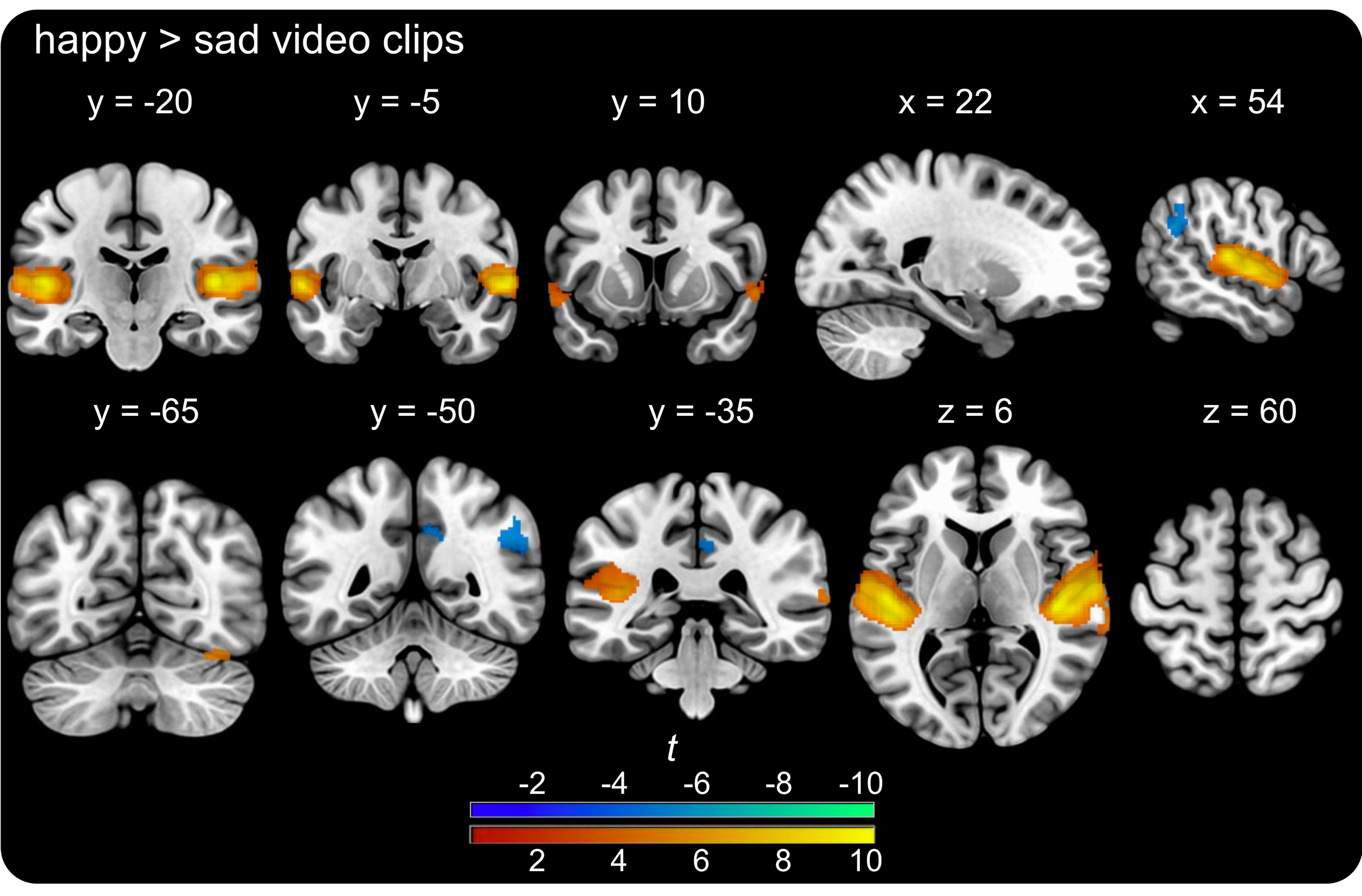


Supplementary Figure 2: Significant brain activations when watching happy compared to sad video clips.
